# Assessing Cognitive and Gut Health in North Carolina’s Rural Indigenous Population via Mobile Clinic

**DOI:** 10.1093/geroni/igaf122.023

**Published:** 2025-12-31

**Authors:** Marianne Chanti-Ketterl, Jada Brooks, Jennifer Jones-Locklear, Heather Whitson

**Affiliations:** Duke University, Durham, North Carolina, United States; The University of North Carolina at Chapel Hill, Chapel Hill, North Carolina, United States; The University of North Carolina at Pembroke, Pembroke, North Carolina, United States; Duke University, Durham, North Carolina, United States

## Abstract

**Objective:**

Alzheimer’s disease disproportionately affects communities of color, including rural North Carolina. These communities experience environmental hazards, limited nutritious food access, and healthcare barriers. To ensure cultural relevance, we collaborated with community members and regional research partners to adapt the Alzheimer’s Gut Microbiome Project protocol (AGMP). Using a mobile clinic, we investigated the relationship between cognitive function, the gut microbiome (GMB), and the metabolome in and around Robeson County, the tribal headquarters of the Lumbee Tribe.

**Methods:**

After tribal IRB approval and informed consent, participants underwent fasting glucose and cholesterol testing via finger prick and biomarker collection through venipuncture. The AGMP protocol for metabolomics and metagenomics was followed. Participants completed cognitive assessments and provided demographic, lifestyle, medical, environmental, and dietary information through structured questionnaires. A home stool collection kit with detailed instructions was provided.

**Results:**

Of the 65 individuals expressing interest, 44 enrolled, and 39 provided both plasma and stool samples. Only three participants did not return stool samples. All participants identified as Indigenous with a mean age of 72 (SD 8); most were women (77%) with a mean of 13 years of education (SD 3). The mean Healthy Eating Index score was 64 (SD 12), and the mean 3-point adjusted Montreal Cognitive Assessment Score was 21 (SD 4). GMB analyses are ongoing.

**Discussion:**

Findings of the association between cognitive function and the GMB will be presented. The high rate of biospecimen collection highlights the effectiveness of our community-centered mobile clinic research approach in a rural area.

